# Hematopoietic stem cell size heterogeneity is not linked to changes in stem cell potential of aged HSCs 

**DOI:** 10.3389/fragi.2025.1596565

**Published:** 2025-05-20

**Authors:** Mehmet Saçma, Ali Hageb, Alex Zadro, Tanja Schuster, Mona Vogel, Karina Eiwen, Vadim Sakk, Hartmut Geiger

**Affiliations:** ^1^ Institute of Molecular Medicine, Ulm University, Ulm, Germany; ^2^ Aging Research Center (ARC), Ulm University, Ulm, Germany

**Keywords:** hematopoietic stem cells, aging, bone marrow niches, cell size and function, regenerative potential, stem cell polarity, stem cell differentiation, hematopoietic aging

## Abstract

Aging is associated with a decline in the function of hematopoietic stem cells (HSCs). This decline in HSC function results in reduced hematologic regenerative capacity and an increased incidence of hematologic disorders. In general, aged HSCs show on average an increase in cell size and a lower frequency of cells polar for protein polarity markers. The size of an HSCs has been proposed to be tightly linked to the potential of the HSCs, with small HSCs showing a higher potential compared to large HSCs. The increase in size of HSCs upon aging may be associated with the reduced potential of aged HSCs. HSCs are located within the bone marrow (BM) in distinct microenvironments called niches. These niches provide critical physical and molecular signals that are essential for HSC self-renewal, proliferation, migration and differentiation. There are multiple types of functional niches, and HSCs within these distinct types of niches show a distinct type of potential. Furthermore, the distribution of HSCs relative to niches changes upon aging. It is not known whether there is a correlation of HSCs size, HSCs polarity and the location of HSCs in distinct types of niches, as might be expected, as all three (size, polarity and position) have been linked to HSC potential. Here we show that in young mice smaller HSCs, which are more myeloid-biased, are preferentially located at central BM niches, including sinusoids and megakaryocytes. In contrast, larger HSCs, which show a bias toward B-lymphoid differentiation, are preferentially located in endosteal BM niches close to arterioles. However, in aged mice, which also contain HSCs of different sizes, there was no correlation between HSC size and localization and potential. Furthermore, within the hematopoietic stem and progenitor cell (HSPC) population, cell size increases as the cells become more limited in their capacity. Notably, we further report that changes in the level of polarity correlate with HSC potential even in aged mice.

## 1 Introduction

Aging has been associated with reduced function of the HSCs, which leads to impaired hematologic regenerative capacity and an increased incidence of blood-related disorders (Rossi et al., 2005; Kamminga et al., 2005; Pang et al., 2017). This decline is a characteristic aspect of aging and contributes to immune system deficiencies and the increased incidence of myeloid malignancies in aged individuals (Kovtonyuk et al., 2016). Although a lot of effort has been given to molecular and metabolic changes in aged HSCs, the relationship between their physical properties, such as cell size, and functional capacity remains less understood (Lengefeld et al., 2021).

HSCs reside in specialized bone marrow (BM) microenvironments named niches, which control their self-renewal, quiescence, and differentiation (Pinho and Frenette, 2019; Morrison and Scadden, 2014). These niches provide distinct physical and molecular signals that are essential for HSC function. Most often, BM niches can be categorized into endosteal niches, rich in osteoblasts and arteriolar vasculature, which are in proximity to the bone surface, and central niches, located deeper within the marrow, with sinusoidal blood vessels and perivascular stromal cells (e.g., CAR cells, LEPR + cells) and megakaryocytes (Pinho and Frenette, 2019). The spatial distribution of HSCs within these niches was reported to change with age, accompanied by changes in niche composition and function (Maryanovich et al., 2018; Saçma et al., 2019; Ho and Méndez-Ferrer, 2020). Endosteal niches, for example, that are rich in arterioles and associated with lymphoid-biased HSCs, undergo significant remodeling during aging, potentially disrupting HSC-niche interactions (Ho and Toro, 2019). Similarly, central niches, characterized by their association with sinusoids and megakaryocytes, exhibit cellular and molecular changes that may influence the behavior of myeloid-biased HSCs (Saçma et al., 2019).

The size of HSCs has been proposed in earlier research to be highly associated with their potential function. Smaller HSCs have been reported to be related to higher regenerative and multilineage differentiation capacities, while larger HSCs are often linked to reduced potential (Lengefeld et al., 2021; Biran et al., 2017; Kumar et al., 2023). This relationship is particularly evident in young HSCs, where size appears to serve as a surrogate marker for functional capacity. However, whether this correlation persists during aging, especially when accounting for the distinctive BM niches, remains unclear. Aging is known to increase the mean size of HSCs, yet it remains to be determined whether this size increase is merely a consequence of aging or whether it directly contributes to the functional decline observed in aged HSCs.

Polarity of HSCs refers to the asymmetric distribution of cellular components, such as proteins and organelles. Loss of polarity in aged HSCs has been linked to increased rates of symetric division. These high levels of symmetric divisions of aged HSCs are tightly linked to the aging-associated dysfunction of HSCs (Amoah et al., 2021; Florian et al., 2018) (Kandi et al., 2021; Florian et al., 2012). The relationship between HSCs polarity, HSCs size and niche localization and its correlation with HSC potential has so far not been determined.

In this study, we investigate the relationship between HSC size, niche localization, and functional potential in both young and old mice. Using advanced imaging techniques, we characterize the spatial distribution of HSCs within diverse BM niches and assess their size as well as their level of polarity. Our results show that young HSCs exhibit size-dependent functional differences that correlate with their niche localization. Specifically, smaller HSCs in central BM niches are more myeloid-biased, while larger HSCs in endosteal BM niches are more lymphoid-biased. However, in old mice, these size dependent differences in HSC potential are no longer observed, suggesting a decoupling of size and function with age.

These findings provide insights into the biological mechanisms underlying hematopoietic stem cells aging. The decoupling of size and potential but not polarity and potential in old HSCs suggests that other factors that affect polarity or are effected by polarity, such as niche interactions, metabolic changes, and epigenetic modifications, play a more dominant role in determining stem cell function during aging (Gao et al., 2025; Nakamura-Ishizu and Suda, 2014; Grigoryan et al., 2018).

## 2 Methods

### 2.1 Whole-mount immunostaining iFAST3D

The iFAST3D imaging protocol allows high-resolution imaging of HSCs and their niche components within intact mouse tissues while preserving their spatial organization (Saçma et al., 2022). Briefly, the protocol starts with sample preparation, where femurs, tibiae or humeri were harvested from mice and fixed in paraformaldehyde to maintain tissue integrity.

The bones were shaved by a cryotome until the BM was fully exposed, ensuring optimal antibody penetration. Immunofluorescence staining followed, involving permeabilization for antibody access, blocking to prevent nonspecific binding and incubation with primary fluorophore-conjugated antibodies targeting HSC markers such as CD150 and CD48, as well as niche components like sinusoids and arterioles. If applicable, secondary antibodies were used for signal amplification. Imaging was performed using confocal laser scanning microscopy for capturing z-stack images to assess the information of the morphology and 3D organization of HSCs and their niches. Data analysis involved image processing to reconstruct 3D visualizations, followed by quantification of HSC size, shape and spatial positioning relative to niche structures, enabling statistical assessment of niche-dependent HSC characteristics. Cell fixation that is part of iFAST3D can affect the cell size, morphology and position, which might affect the correct quantiative determination of parameters. We used an even further optimized iFAST3D protocol from Saçma et al., 2022 that is designed to even better preserve cell morphology and tissue architecture, by minimizing the use of chemical solvents, incubation time and mechanical stress.

### 2.2 CellDetail analysis

To assess the size and polarity of FACS isolated single HSCs, specifically focusing on Tubulin and Cdc42, we employed a protocol adapted from the recent published methodology CellDetail (Schuster et al., 2024). Briefly, this approach involves immunofluorescence staining of FACS-isolated and fixed HSCs, followed by epifluorescence or confocal microscopy to capture subcellular high-resolution images. Next, the spatial distribution of Tubulin and Cdc42 within the cells was analyzed using the specialized image analysis software CellDetail, which calculates polarity values based on fluorescence intensity distributions. Further, this method allowed for precise measurement of cell size and the valuation of polarity status in both young and aged isolated HSCs.

### 2.3 Flow cytometry-based size analysis

The absolute size of individual HSPC populations from young and aged mice was determined by flow cytometry using forward scatter (FSC) measurements calibrated with reference size beads. FSC-W histograms were created using 7 µm, 10 µm, and 16 µm reference beads from the SPHEROTM Flow Cytometry Particle Size Standard Kit.

A calibration plot was constructed to establish a standard curve with FSC measurements proportional to the bead diameter and a significant R^2^ value, using the formula for cell size calculation. Bead-referenced average cell sizes were determined for LT-HSCs, ST-HSCs, and LMPPs isolated from both endosteal and central BM of young and aged mice.

## 3 Results

### 3.1 HSCs in distinct niches within the BM are different in size

**FIGURE 1:**
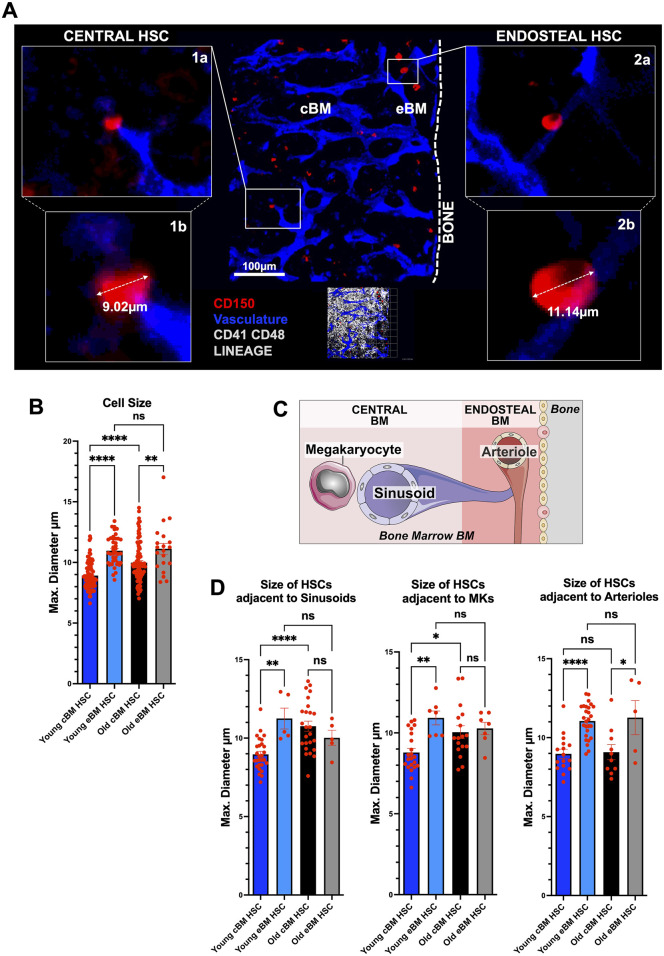
HSC in distinct niches within the BM are different in size. **(A)** Extended focus projection of whole-mount confocal images from a femur of a young C57BL/6 WT mouse. Shown are vasculature (blue, CD31 CD144), bone surface (dashed line denote the endosteum) and the central and endosteal BM (cBM, ≥50 µm from the endosteum, and eBM, <50 µm from the endosteum). 500 µm × 500 µm. HSCs are CD150^+^ (red) and negative for the other hematopoietic markers (CD41, CD48 and lineage, grey). Zoom-in 1a,b (one xy layer): HSC located in the central BM with max. diameter; zoom-in 2a,b (one xy layer): endosteal HSCs with max. diameter. **(B)** Maximum cell diameter of CD150^+^CD41^-^CD48^−^LIN^-^ HSCs *in situ* from eBM and cBM in young (8–16-weeks-old) and old (>old 80-weeks-old) long bones **(C)** General scheme showing bone and bone marrow with endosteal and central parts and preferential location of arterioles, sinusoids and megakaryocytes. **(D)** Maximum cell diameter of HSCs from eBM and cBM in young and old long bones adjacent (<10 µm) to sinusoids (young: n = 28 HSCs in cBM, n = 5 HSCs in eBM; old: n = 25 HSCs in cBM, n = 5 HSCs in cBM), adjacent to megakaryocytes (Young: n = 22 HSCs in cBM, n = 7 HSCs in eBM; old: n = 17 HSCs in cBM, n = 7 HSCs in cBM) or adjacent to arterioles (Young: n = 16 HSCs in cBM, n = 27 HSCs in eBM; old: n = 10 HSCs in cBM, n = 5 HSCs in cBM). For **(B)** and **(D)** in total young: n = 41 HSCs in eBM, n = 86 HSCs in cBM, from n = 31 3D-images, n = 7 mice; old: n = 20 HSCs in eBM, n = 95 HSCs in cBM, from n = 11 3D-images, n = 4 mice; three to four independent experiments. Data are mean ± s.e.m. One-way-ANOVA-test. ns = not significant, *P < 0.05, **P < 0.01, ****P < 0.0001.

Using 3D confocal microscopy, we first confirmed that HSCs (LSK, CD34^-^, Flk2^-^) from BM of aged mice (>80 weeks old) were not only on average larger (increase in volume) than HSCs from young mice (8–20 weeks old), but aged HSCs also showed on average a larger (increase in volume) nucleus (Supplementary Figure S1A,B), as previously published (Grigoryan et al., 2018). To determine the size of HSCs in distinct niches in BM of long bones of C57BL/6 mice, we next used the iFAST3D method for IF staining of HSCs within BM^11^ (Supplementary Figure S1C,D). In young as well as in aged mice, HSCs (CD150^+^CD41^-^CD48^−^LIN^-^) that were located in the central BM were on average smaller compared to HSCs located in endosteal niches (Figures 1A,B). The smaller HSCs in the central BM were found preferentially adjacent to central sinusoids and megakaryocytes, whereas larger HSCs at the endosteum were in most cases positioned adjacent endosteal arterioles (Figures 1C,D; Supplementary Figures S1C,S1D). HSCs size is therefore distinct among HSCs from distinct regions and niches in BM. In aged mice, the relative difference in size of HSCs between the two niches was maintained, while old HSCs were on average larger than young HSCs (Figures 1A–D). Interestingly, young periarteriolar HSCs in endosteal BM were though on average larger than aged periarteriolar HSCs in central BM (Figure 1D). Notably, the proportions of HSCs adjacent to sinusoids and megakaryocytes in the central BM (cBM) were significantly higher than those in the endosteal BM (eBM) in both young and aged mice (HSCs adjacent to sinusoids: young cBM 85%, young eBM 15%, old cBM 86%, old eBM 14%; HSCs adjacent to megakaryocytes: young cBM 76%, young eBM 24%, old cBM 71%, old eBM 29%). In contrast, the proportion of HSCs adjacent to arterioles was higher in eBM than in cBM only in young mice (HSCs adjacent to arterioles: young cBM 37%, young eBM 63%, old cBM 67%, old eBM 33%, Supplementary Figure S1E). These results suggest a shift, in which fewer HSCs remain in the eBM in aged mice, likely due to age-related remodeling of BM niches, affecting either accessibility or function. (Saçma et al., 2019; Maryanovich et al., 2018; Ho and Toro, 2019). The absolute size of distinct HSPCs populations from distinct BM niches from young and aged mice was determined by flow cytometry (forward scatter, FSC) calibrated with reference size beads (Supplementary Figures S2A,S2B). HSCs from central bone marrow (sinusoidal or megakaryocytic niches) were also with respect to their absolute size smaller than HSCs located at endosteal niches, while in general the absolute size of HSCs from aged mice was increased in both types of niches when compared to young HSCs with the respective niche localization (Supplementary Figure S2C). Also in absolute size, young HSCs from endostal niches were larger than aged HSCs from central niches. Interestingly, short-term repopulating HSCs (ST-HSCs), and especially lympho-myeloid primed progenitor cells (LMPPs), which are only slightly more differentiated compared to HSCs, show already a strong increase in overall cell size (young and aged) compared to their respective HSCs (Supplementary Figure S2C).

Similarly, when differentiating between small, medium and large cells based on forward scatter (Supplementary Figure S2D), large LMPP and large LSK cells were both significantly larger compared to HSCs, irrespective of age or niche position (Supplementary Figure S2E). Progenitors cells show on average a high turnover and are thus frequently actively cycling (Rossi et al., 2007), and active cells may need to maintain a large cell size to allow for cell cycle progression and subsequent division (Conlon and Raff, 1999), which might explain the relatively large size of, for example, LMPPs. Finally, HSCs outside their native microenvironment like in our flow experiments have a smaller cell size compared to HSCs in their native microenvironment in BM *in situ* (Figure 1B; Supplementary Figure S2C).

### 3.2 Differences in cell size of aged HSCs from distinct BM regions do not correlate with potential

**FIGURE 2:**
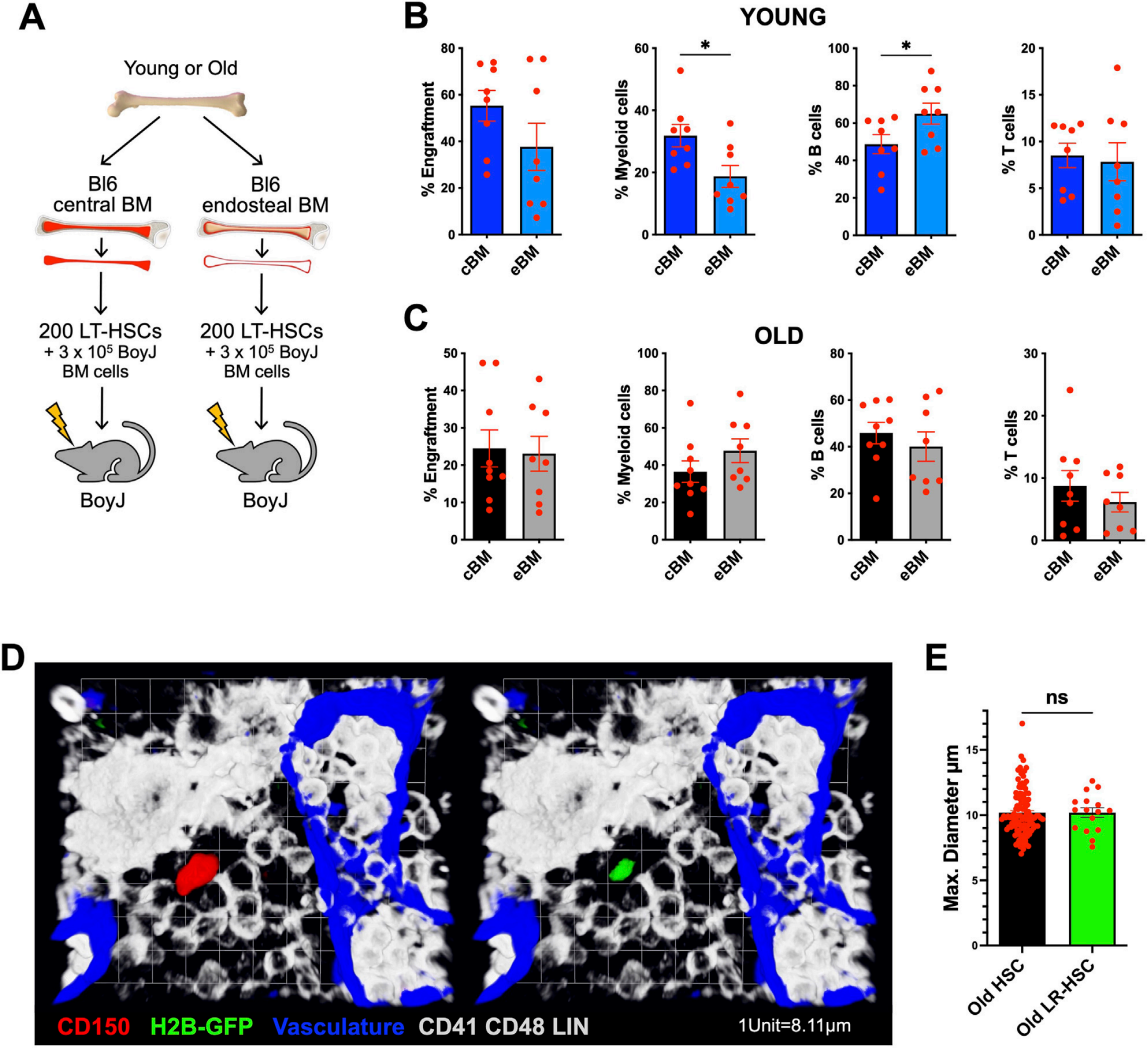
Differences in cell size of aged HSCs from distinct BM regions do not correlate with a difference in potential. **(A)** Experimental setup. 200 LT-HSCs from young and old BL6 mice from central or endosteal BM were transplanted into sub-lethally irradiated BoyJ mice alongside 3 × 10^6^ BoyJ competitor BM cells. Donor chimerism was determined in PB at 20 weeks post-transplantation. **(B, C)** Donor chimerism (percent of overall engraftment), and percentages of myeloid cells, B-cells or T-cells among all donor-derived cells in PB in recipient animals transplanted with either endosteal and central donor HSCs from **(B)** young or **(C)** old recipients at 20 weeks post-transplantation (n = 8-9 recipient animals per group, n = 9 young donor animals and n = 3 old donor animals in two independent batches). **(D)** 3D reconstruction of whole-mount images. GFP^+^CD150^+^CD41^−^CD48^−^Lin^−^ label-retaining-HSC (nucleus green) in proximity to sinusoidal vessels (blue) within old SCL-tTAxH2B-GFP BM. **(E)** Maximum cell diameter of old HSCs and old label-retaining (nucleus green)-HSCs *in situ.* (old: n = 115 HSCs, from n = 11 3D-images, n = 4 mice; old label-retaining: n = 18 HSCs, from n = 16 3D-images, n = 6 mice). Data are mean ± s.e.m. Unpaired t-test. ns = not significant, *P < 0.05.

To compare the stem cell and multilineage reconstitution potential of HSCs that are located in the 2 distinct BM regions and which are therefore distinct in their average size, we competitively transplanted 200 LT-HSCs from either central (smaller HSCs) or endosteal (larger HSCs) BM alongside 3 × 10^5^ BoyJ competitor BM cells into sublethally irradiated BoyJ mice (Figure 2A). Endosteal HSCs from young mice (larger) showed a bias towards B-lymphoid differentiation, whereas HSCs from central BM (smaller) of young mice were more myeloid-biased (Figure 2B). The on average larger size of HSCs located at the endosteum (Figure 1D) is thus linked to a preferential lymphoid reconstitution potential, and the on average smaller sized central BM HSCs are linked to preferential myeloid-biased reconstitution. These data support previous suggestions that lymphoid-biased HSCs are preferentially located in periarteriolar niches at the endosteum and myeloid-biased HSCs close to perisinusoidal MKs in the central BM (Pinho et al., 2018). In contrast, HSCs from old mice from both the endosteal (larger size) and the central part (smaller size) of the BM show a similar level of engraftment and multilineage reconstitution (Figure 2C). As aged HSCs located at the endosteum are on average larger than HSCs located in the central BM, differences in cell size among aged HSCs in the endosteal and central regions do not correlate with differences in potential. Interestingly, label-retaining HSCs (Figure 2D), which represent the most quiescent HSC subpopulation with the highest regenerative potential in old mice and are thus functionally more young-like (Saçma et al., 2019), exhibit a cell size similar to that of all old HSCs (Figure 2E). These findings suggest that size alone is insufficient to predict HSC function in aged mice.

### 3.3 Polarity of aged HSCs from distinct BM regions correlates with HSC potential

**FIGURE 3:**
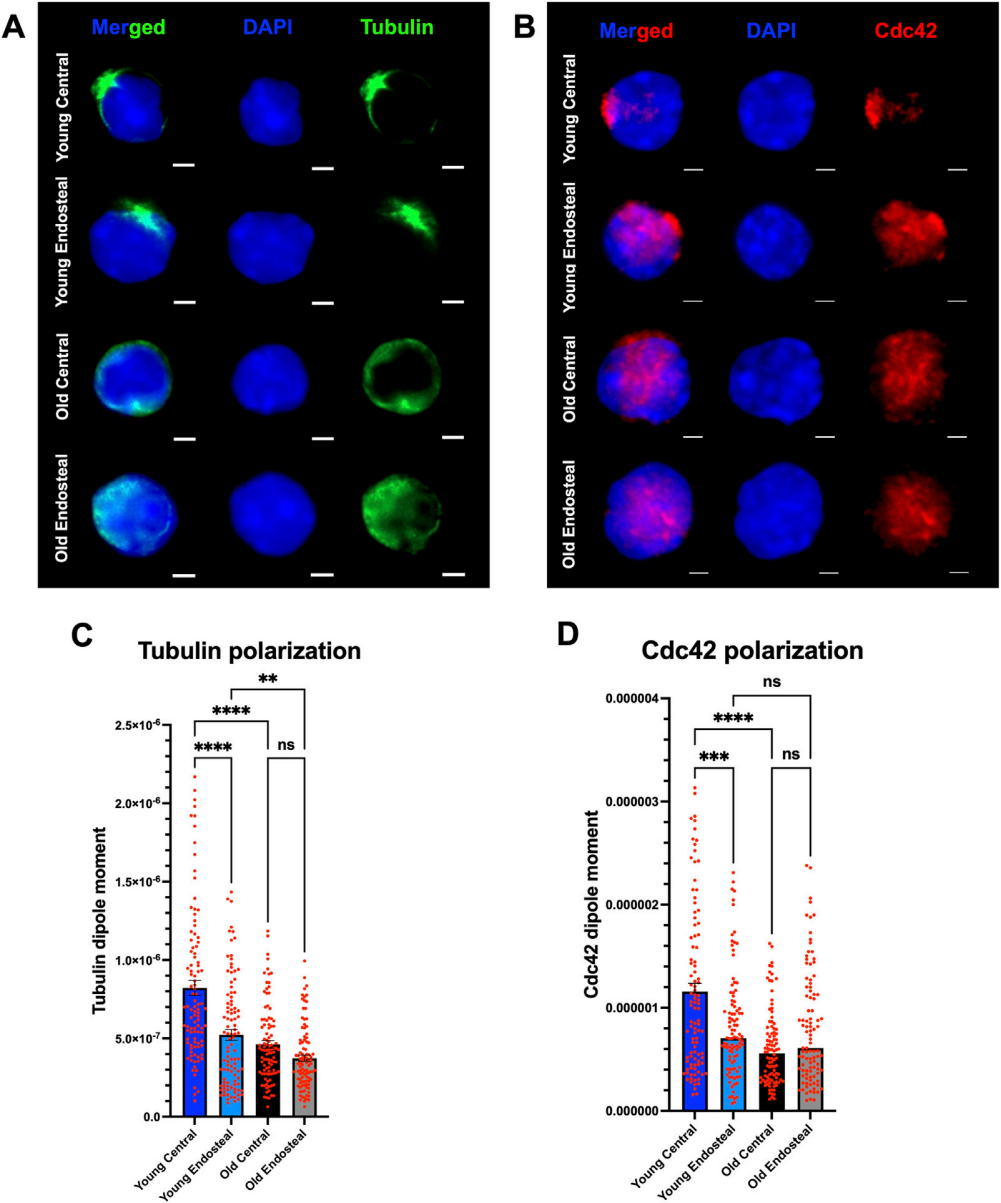
Cell polarity is a consistent parameter for stem cell capacity. **(A)** Representative single-cell immunofluorescence images showing Tubulin (green) and nuclei (DAPI, blue) in young and old central and endosteal LT-HSCs. Scale bar = 2 μm. **(B)** Representative single-cell immunofluorescence images showing Cdc42 (red) and nuclei (DAPI, blue) in young and old central and endosteal LT-HSCs. Scale bar = 2 μm. **(C)** Tubulin polarization assessed by Tubulin intensity dipole moments from young and old central and endosteal LT-HSCs. **(D)** Cdc42 polarization assessed by Cdc42 intensity dipole moments from young and old central and endosteal LT-HSCs. n = 100 LT-HSC per group, n = 3 experiments, n = 9 young (3 pooled per experiment) and n = 3 old mice. Data represent mean ± s.e.m and the statistical significance was assessed by ordinary one-way-ANOVA-test. ns = not significant, **P < 0.01, ***P < 0.001, ****P < 0.0001.

In general, among aged HSCs there is a significantly reduced frequency of HSCs polar for distinct types of polarity markers. This loss of cell polarity is tighlty linked to the aging associated dysfunction of aged HSCs (Florian et al., 2012). We thus investigated the extent to which the frequency of aged polar HSCs is distinct among central or endosteal HSCs. Single-cell IF analyses of young and old central and endosteal LT-HSCs (Figures 3A,B) revealed that young central HSCs are more polar (cells with a higher dipole moment) for the established polarity markers tubulin and Cdc42 compared to endosteal young HSCs, and especially to aged HSCs (Figures 3C,D). Among aged endosteal or central HSCs, there was no difference in the frequency of polar HSCs. The level of polarity does correlate better than the cell size to the potential of HSCs determind in the transplantation experiments (Figures 2A–C).

## 4 Discussion

In conclusion, our results show that young or aged HSCs within distinct niches show distinct cell sizes, with endosteal HSCs being larger than central HSCs. Second, they support previous findings demonstrating, a general increase in the average size of HSCs upon aging (Lengefeld et al., 2021; Biran et al., 2017; Kumar et al., 2023). Third, they demonstrate that young HSCs differ in multilineage differentiation potential (smaller myeloid-, larger lymphoid biased). Fourth, the difference in potential among aged HSCs does not correlate with the difference in cell size of aged HSCs. At least among aged HSCs, cell size does not inform on stem cell potential (Lengefeld et al., 2021). Five, the level of polarity among young and aged HSCs from distinct BM niches correlates with HSC potential. Our findings describe HSCs and niches from C57BL/6 mice. Age-related niche dynamics may differ in other strains or in humans.

The distinct size of HSCs in different BM niches might be influenced by specific interactions between stem cells and their respective microenvironment, such as regulation of cell division, differentiation and self-renewal. Also, different physical and chemical properties of the different niches, such as stiffness, pressure and adhesion, and the availability of nutrients, oxygen and the metabolic status might influence cell size. Further experiments will be necessary to test these hypotheses. We have, for example, previously demonstrated an influence of extracellular pH on the size of HSCs (Kumar et al., 2023). The fact that among aged HSC in distinct BM niches size is not linked to distinct potentials suggests that other parameters like polarity/apolarity for cytoplasmic or nuclear proteins might be better suited to predict potential of aged HSCs (Saçma et al., 2019; Maryanovich et al., 2018; Ho and Toro, 2019). We can only specualte on likely mechanisms underlying this decoupling of HSC size and function with aging, and additional experimental work will need to be performed to obtain more insight. Size in aged HSCs might be determined by the level of niche support or changes in metabolism that are not directly linked to changes in polarity/potential like in young HSCs.

The level of activity of the small RhoGTPase Cdc42 regulates polarity in HSCs (Florian et al., 2012). While this work correlates polarity to HSC function, the underlying mechanisms that link polarity to HSCs function are not known. Further experiments are needed to understand potential mechanisms downstream of polarity that affect function, inlcuding metabolic changes such as increased glycolytic reliance affected by the 3D distribution of proteins, or epigenetic drifts induced by an altered polar distribution of proteins in HSCs (Bartram and Filippi, 2022; Florian et al., 2012; Amoah et al., 2021; Kramer and Challen, 2017).

## Data Availability

The original contributions presented in the study are included in the article/Supplementary Material, further inquiries can be directed to the corresponding authors.

## Supplementary material

SUPPLEMENTARY FIGURE S1
**(A)** Left: Representative 3D reconstruction with segmentation of single-cell IF confocal images of young and old LSK CD34^−^ Flk2^−^ LT-HSCs. Right: Cell volume of young and old LT-HSCs. (young: n = 80 HSCs, from n = 9 3D-images, n = 4 bones; old: n = 250 HSCs, from n = 9 3D-images, n = 6 bones). **(B)** Left: Representative 3D reconstruction of single-cell IF confocal images by DAPI signal of young and old LSK CD34^−^ Flk2^−^ LT-HSCs. Right: nuclear volume of young and old LT-HSC. (young: n = 44 HSCs, from n = 5 3D-images, n = 3 bones; old: n = 57 HSCs, from n = 3 3D-images, n = 2 bones). Data are mean ± s.e.m. Unpaired t-test. ns = not significant, ***P* < 0.01. **(C)** Upper part: Representative confocal images of an HSC *in situ* adjacent to a sinusoid in cBM in a young femur. Lower part: Representative z-stacked confocal images of an HSC *in situ* adjacent to a megakaryocyte in the cBM of a young femur **(D)** Representative confocal image of HSCs *in situ* adjacent to an arteriole in eBM in a young femur (upper image z-stacked, lower image one xy layer). **(E)** Proportions of HSCs adjacent to sinusoids, megakaryocytes (MKs), and arterioles in endosteal and central BM compartments in young and old mice.

SUPPLEMENTARY FIGURE S2
**(A)** FSC-W histogram of 7µm, 10µm and 16 µm sized reference beads from the SPHERO^TM^ Flow Cytometry Particle Size Standard Kit. **(B)** Calibration plot for standard curve generation using the reference sizing beads. FSC measurement are proportional to the diameter of the beads with a significant R^2^ value and the formula for cell size calculation: y = 5561.9*x + 49249. **(C)** Bead-referenced mean cell size of LT-HSCs, ST-HSCs, LMPPs isolated from young and old endosteal and central BM. **(D)** Representative FACS contour plot (left) and histogram (middle) of LSK CD34^−^Flk2^−^ LT-HSCs isolated from central BM of young mice. For middle cell size cell determination, a gate of 10k FSC-A units was positioned symmetrically around the maximum value in the contour plot (M). Small (S) and large (L) cell gates were positioned at a distance of 4k FSC-A units from the M gate in the histograms. LMPP FACS histogram (right) with cell size gates. **(E)** Fold expansion in size of large LSK cells and LMPPs isolated from endosteal and central BM of young and old mouse bones compared to young LT-HSC counterparts (n = 5–6 experiments, n = 15–18 young (3 pooled per experiment) and n = 5–6 old mice). Data are mean ± s.e.m.
