# Supplementary material for: Hematopoietic stem cell size heterogeneity is not linked to changes in stem cell potential of aged HSCs
Source: Front Aging. 2025 May 20;6:1596565. doi: 10.3389/fragi.2025.1596565 (PMC12129979; doi:10.3389/fragi.2025.1596565)
Supplement: Supplementary file 1 [file DataSheet1.pdf]

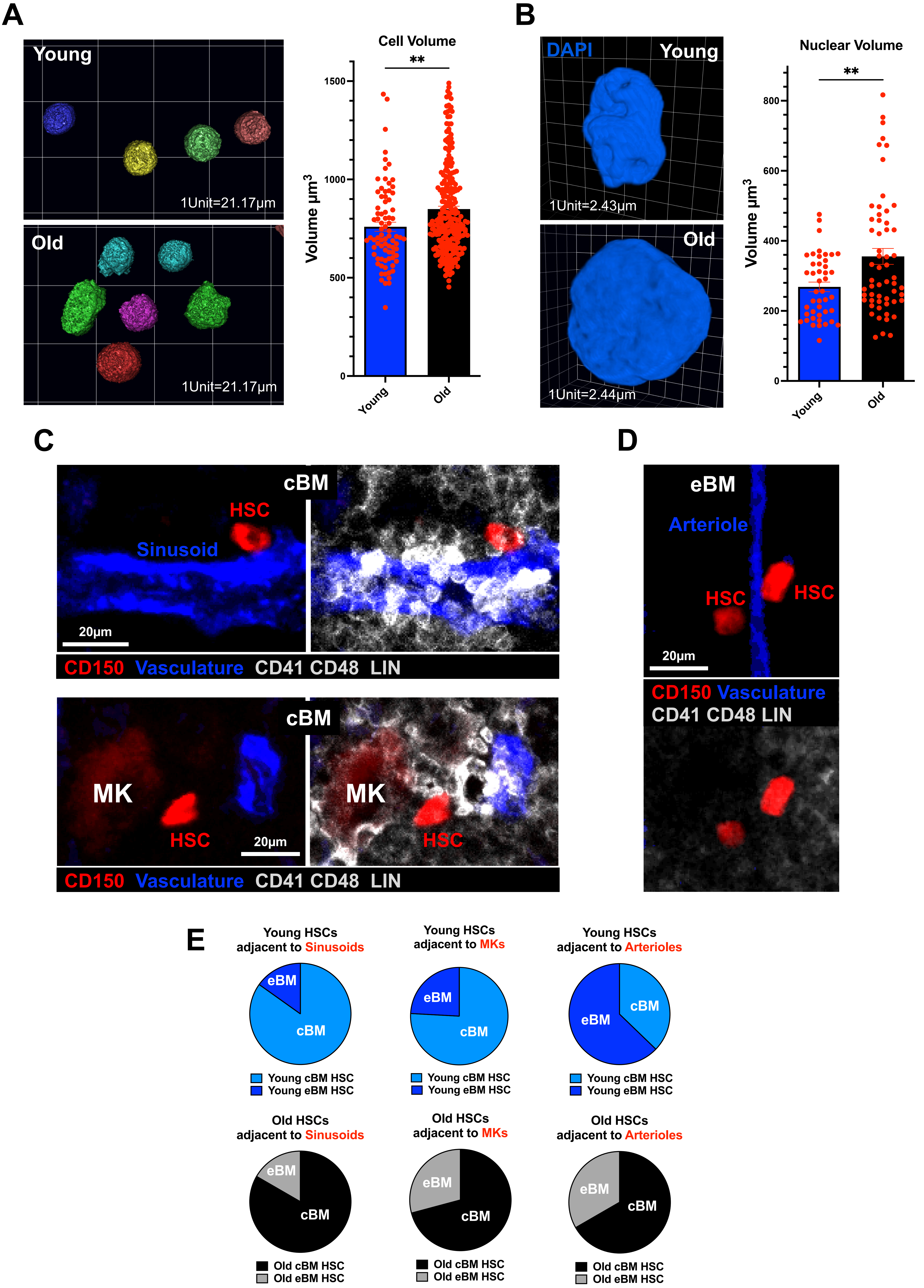
A
Young
1Unit=21.17μm
Old
1Unit=21.17μm
Cell Volume
**
Volume μm3
1500
1000
500
0
Young
Old
B
DAPI
Young
1Unit=2.43μm
Old
1Unit=2.44μm
Nuclear Volume
**
Volume μm3
800
600
400
200
0
Young
Old
C
cBM
HSC
Sinusoid
20μm
CD150 Vasculature CD41 CD48 LIN
cBM
MK
HSC
20μm
MK
HSC
CD150 Vasculature CD41 CD48 LIN
D
eBM
Arteriole
HSC
HSC
20μm
CD150 Vasculature
CD41 CD48 LIN
E
Young HSCs adjacent to Sinusoids
eBM
cBM
Young cBM HSC
Young eBM HSC
Young HSCs adjacent to MKs
eBM
cBM
Young cBM HSC
Young eBM HSC
Young HSCs adjacent to Arterioles
cBM
eBM
Young cBM HSC
Young eBM HSC
Old HSCs adjacent to Sinusoids
eBM
cBM
Old cBM HSC
Old eBM HSC
Old HSCs adjacent to MKs
eBM
cBM
Old cBM HSC
Old eBM HSC
Old HSCs adjacent to Arterioles
eBM
cBM
Old cBM HSC
Old eBM HSC

SFigure 1

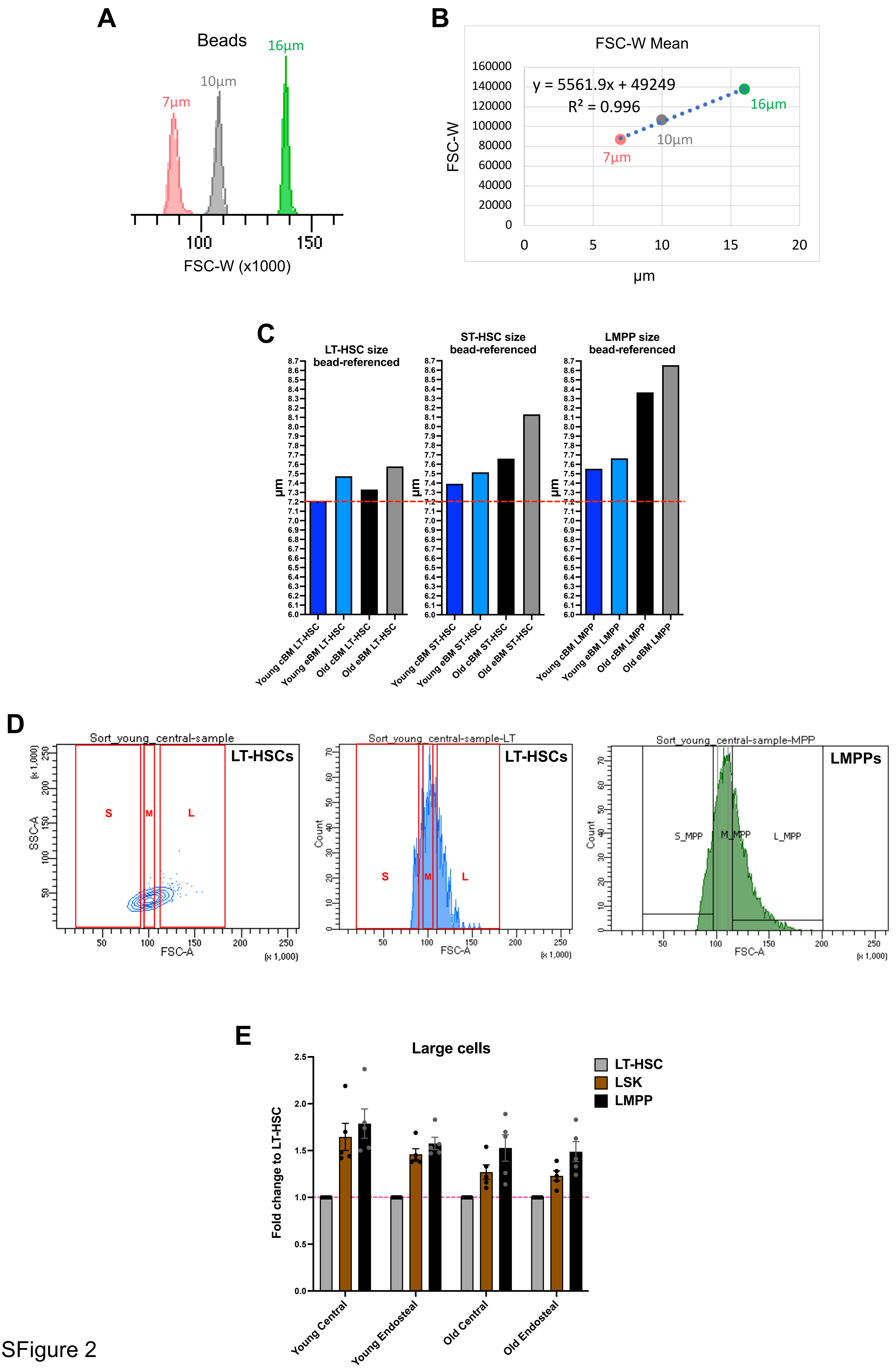
A
Beads
7µm
10µm
16µm
100
150
FSC-W (x1000)
B
FSC-W Mean
y = 5561.9x + 49249
R² = 0.996
FSC-W
µm
C
LT-HSC size bead-referenced
ST-HSC size bead-referenced
LMPP size bead-referenced
µm
Young cBM LT-HSC
Young eBM LT-HSC
Old cBM LT-HSC
Old eBM LT-HSC
Young cBM ST-HSC
Young eBM ST-HSC
Old cBM ST-HSC
Old eBM ST-HSC
Young cBM LMPP
Young eBM LMPP
Old cBM LMPP
Old eBM LMPP
D
Sort_young_central-sample
Sort_young_central-sample-LT
Sort_young_central-sample-MPP
LT-HSCs
LMPPs
S
M
L
S_MPP
M_MPP
L_MPP
SSC-A
Count
FSC-A
(x 1,000)
E
Large cells
LT-HSC
LSK
LMPP
Fold change to LT-HSC
Young Central
Young Endosteal
Old Central
Old Endosteal

SFigure 2
